# The effect of the Bilateral Integration exercise program on the cognitive functioning of pupils with moderate intellectual disabilities

**DOI:** 10.3389/fpsyt.2024.1409061

**Published:** 2024-10-22

**Authors:** Magdalena Koper, Magdalena Lewandowska, Małgorzata Rękosiewicz

**Affiliations:** ^1^ Department of Adapted Physical Activity, Poznan University of Physical Education, Poznan, Poland; ^2^ Calculation Centre, Poznan University of Physical Education, Poznan, Poland; ^3^ Faculty of Psychology and Cognitive Sciences, Adam Mickiewicz University in Poznań, Poznan, Poland

**Keywords:** intellectual disability, physical activity, bilateral integration activities/exercises, executive function, cognitive development

## Abstract

**Introduction:**

Children with intellectual disability tend to exhibit lower performance in fundamental movement skills, such as locomotor skills, object control skills, and balance, compared to their typically developing peers. Evidence suggests that physical activity programs tailored for individuals with intellectual disabilities can positively influence the development of their motor skills. Similar to typically developing children, physical activity in children with intellectual disabilities stimulates not only physical development, but also brain function, resulting in cognitive benefits. However, the extent of physical activity’s impact on the cognitive functioning of this population remains insufficiently explored. The aim of our study was to assess the effectiveness of a motor exercise program based on the Bilateral Integration method implemented for pupils with moderate intellectual disabilities.

**Methods:**

The sample consisted of 27 pupils with moderate intellectual disability (13 in the intervention group and 14 in the control group) from two special schools in Poland. Pupils in the intervention group participated in a modified version of the Bilateral Integration School Program - a 26-week physical activity program that included both individual and group sessions. The assessment of cognitive functioning, using the Fifth Edition of the Stanford–Binet Intelligence Subtests, was conducted over three time points in both groups: a pre-test before the intervention (T1), a post-test after the intervention phase end (T2) and a follow-up assessment three months after the post-test (T3).

**Results:**

Significant effects of the intervention compared to the control group were observed in certain cognitive variables. Repeated measures ANOVA revealed a significant group-by-time interaction for the Nonverbal Visual-Spatial Processing score, Nonverbal Working Memory score, as well as Verbal Fluid Reasoning.

**Discussion:**

The original program based on the Bilateral Integration method proved effective for pupils with moderate intellectual disability. Preliminary results indicate improvements in cognition, particularly in working memory and visual-spatial processing. Further studies are necessary to assess the program’s efficacy comprehensively.

## 1 Introduction

Intellectual disability is characterized by a significant reduction in general intellectual functioning, accompanied by limitations in adaptive behavior. This lifelong condition manifests during developmental years and is classified under neurodevelopmental disorders in the ICD-11. Recent meta-analysis estimates the prevalence of intellectual disability at 10.37 per 1,000 individuals (1). However, studies focusing on children and adolescents report a higher prevalence of 18.30 per 1,000. The distribution of severity levels among individuals with intellectual disability is approximately 85% for mild, 10% for moderate, 4% for severe, and 1% for profound cases (2).

The diagnostic criteria for intellectual disability include: (1) a significantly reduced intellectual level, typically measured as an IQ below 70 which results in impairments across various cognitive processes, and (2) limitations in adaptive behavior. Literature often highlights that children with intellectual disability exhibit notable deficits in various cognitive functions, such as executive functions, attention, memory, and language (3). However, research of executive functions presents a varied picture. Executive function is conceptualized as a set of higher cognitive skills necessary for daily activities and abilities, including planning, organizing, cognitive flexibility, working memory and self-regulation. Some studies suggest that children with intellectual disability have a substantial deficit in executive functions compared to typically developing peers matched for chronological age. Furthermore, among children with intellectual disabilities, those with a mild level of disability generally perform better on tasks assessing executive functions than those with a moderate intellectual disability (4–7). This indicates that executive function performance may be related to the level of intellectual disability. Comparative studies of children with intellectual disabilities, matched for mental age to typically developing peers, show that the performance of children with intellectual disabilities either matches or is slightly lower than that of their typically developing counterparts (8–11). Overall, children with intellectual disabilities exhibit a heterogeneous range of cognitive deficits (12).

Children with intellectual disability often face challenges with verbal executive-loaded working memory, as well as with inhibition and planning (5). Regarding working memory capacity, both phonological working memory (the ability to store and manipulate auditory information) and visual-spatial working memory (the ability to store and manipulate visual information) are typically impaired (13, 14). Additionally, these children have an increased risk of difficulties with visual-spatial information processing, which is often associated with a lower IQ (15, 16).

There is some empirical evidence suggesting that individuals with intellectual disability also face movement and motor limitations. A systematic review of the literature indicated that children with intellectual disability lack mastery of fundamental movement skills and exhibit deficits or developmental delays in these areas. They generally demonstrate significantly lower performance in fundamental movement skills, such as locomotor skills, object control skills, and balance skills, compared to typically developing children (17). The review included studies with participants with borderline, mild, or moderate intellectual disabilities. Those with profound intellectual or multiple disabilities are at an even greater risk of deficits due to frequent periods of motoric inactivity, which limits their opportunities to train and improve movement skills (18).

Carefully designed and targeted physical activity programs for people with intellectual disabilities can positively influence the development of their basic motor skills (19). Ma et al. (20) found that six interventions programs used in their study - fitness exercise, combined strength and proprioceptive training, dual-task functional exercises, trampoline exercise, hippotherapy, and core strength training - were effective in improving balance ability in children. Additionally, combined physio-hemsball training was shown to enhance postural control, dynamic balance, and static balance among children with intellectual disabilities (21). However, research typically focuses on participants with mild intellectual disabilities, with a notable lack of programs designed for individuals with more severe levels of disability (22).

It is acknowledged that physical activity not only leads to positive outcomes in physical health but also benefits mental health among individuals with intellectual disabilities, such as increased quality of life indicators among adolescents with intellectual disability (23). Additionally, several studies investigating the effects of physical activity on cognition in children have found that exercise can lead to cognitive improvements. For instance, in a longitudinal study examining children with intellectual disabilities and borderline intellectual disabilities found that skill-related physical fitness was associated with improved inhibition and cognitive flexibility (24). Furthermore, deficits in motor skills and executive functions have been shown to be interrelated (25). Specifically, Wuang et al. (26) demonstrated a positive relationship between fundamental movement skills performance and processing speed as well as verbal comprehension. Therefore, early interventions that enhance both motor and cognitive development in children with intellectual disabilities are recommended.

Some research has indicated that, regarding the effect of physical activity on cognitive functioning, not only the intensity of physical activity but also regularity/frequency and type of exercises are crucial (23, 26, 27). Findings from a review highlighted that cognitively engaging exercises have a more significant impact on children’s executive functioning compared to non-cognitively engaging exercises (29). Schmidt et al. (30) found that physical activities requiring substantial cognitive engagement had a more beneficial impact on executive functioning. They compared three conditions: (1) non-active, (2) aerobic activities with low cognitive engagement and (3) team games with high cognitive engagement. Additionally, a novel approach to physical education, the Better Movers and Thinkers program, has demonstrated positive effects on coordination and executive functions in typically developing children (28, 31). In this study, the effects of 16-week intervention (2 hours of physical education each week) using the Better Movers and Thinkers approach were compared with those of the control group, which received the standard physical education curriculum.

Research has shown that, aside from the well-established links between aerobic fitness and executive functions, skill-related physical fitness may be an even stronger predictor of cognitive development in typically developing children. It has been hypothesized that, in addition to aerobic mechanisms, learning and developmental mechanisms are crucial, as skill-related movements provide learning experiences that support cognitive growth (32). Skill-related physical fitness includes components of physical fitness associated with improved performance in sports and motor skills, with coordination and agility being key features (33). Coordination refers to the ability to use the senses together with body parts to perform motor tasks, while agility is the ability to change the body’s position rapidly with speed and accuracy (33). Research has also shown that various brain structures play a critical role in both skill-related movements and executive functions (34, 35). In typically developing children, a strong relationships between complex motor skills and higher-order cognitive functions has been demonstrated (36). In individuals with intellectual disability, such as adolescents with Down syndrome, a positive relationship has been found between planning ability and manual dexterity (37).

Research on able-bodied individuals has demonstrated that engaging in physical activity is believed to enhance cognition through changes in the brain’s structure and function. A systematic review by Singh et al. (38) highlights that the positive effects of physical activity on cognition can be attributed to its role in promoting neurogenesis, angiogenesis, and improving the central nervous system’s metabolism. Moreover, regular physical activity is thought to increase the levels of specific growth factors, including brain-derived neurotrophic factor (BDNF), which plays a critical role in the development, modification, and maintenance of brain structures and functions, directly influencing learning and memory processes (39, 40).

As previously discussed, the literature has established connections between interventions targeting executive function skills and improvements in cognition among able-bodied children. It is expected that skill-related physical fitness, which facilitates the smooth and accurate performance of motor tasks, will also be positively associated with executive functions in children with intellectual disabilities. Therefore, the primary aim of the present study was to investigate the impact of bilateral integration exercise program on the cognitive functioning of pupils with moderate intellectual disabilities.

## 2 Methods

### 2.1 Participants and procedures

Our research involved pupils participating in the project entitled Original model of supporting pupils with special educational needs based on the school program of the Bilateral Integration method (Grant EOG/21/K4/W/0086), funded under the Educational Program financed by EEA FM for the years 2014 - 2021, Component IV “EDUCATION” PROGRAM. The project was implemented from March 2022 to December 2023. Pupils were recruited from two special education centers/schools involved in the project for the intervention and control groups, respectively. The project included all pupils attending primary school classes in these institutions during the 2022/23 school year who agreed to participate in the project and met the inclusion criteria. Ultimately, 52 pupils were qualified (26 in the intervention group and 26 in the control group) all of whom had moderate intellectual disability and without co-existing disorders (verified through current certificates of special educational needs issued by public psychological and pedagogical counseling centers). The participants ranged in age from 8 to 20 years, in accordance with the Education Law in force in Poland. The parents/guardians of the pupils qualified for the project were informed about the goals and organization of the project activities and provided written informed consent for their child’s participation.

**Figure 1:**
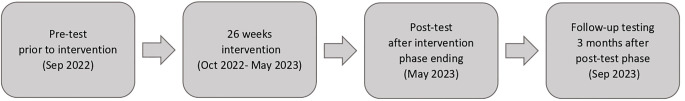
Chronology of pre-test (T1), intervention, post-test (T2) and follow-up testing (T3).

The project included: 1) the development and implementation of an innovative support model based on the Bilateral Integration method (BI), conducted through exercise classes following original lesson plans, 2) training of staff responsible for conducting classes with the pupils, and 3) an assessment of the effectiveness of the intervention. A natural experiment design was used to evaluate the effectiveness of the intervention. All pupils involved in the project followed the same core curriculum and had no prior exposure to the BI method. Pupils in the intervention group participated in additional extracurricular activities as part of the intervention program (a 26-week BI program implemented during the school year), while pupils in the control group continued their regular school routine without any modifications during T1, T2, and T3. Between T2 and T3, pupils from both study groups had a summer break. The project was implemented in four phases, as shown in **Figure 1**. The research, which included the assessment of cognitive functioning in pupils with moderate intellectual disabilities, was conducted across three terms in both the intervention and control groups: the first term – at the beginning of the school year, before the start of the intervention (pre-test); the second – at the end of the school year, at the end of the intervention (post-test); and the third – after the summer break, three months after the end of the intervention (follow-up testing).

For the purposes of this article, only pupils who had complete results across all three test terms and who were aged 18 years or younger were included in the analysis. Additionally, the selection of pupils from both the intervention and control groups was conducted with the goal of ensuring homogeneity in terms of sociodemographic variables between the groups, thus minimizing potential environmental influences (see **Table 1**). Consequently, the final sample consisted of 13 pupils in the intervention group and 14 pupils in the control group.

**Table 1:** Sociodemographic characteristic in the intervention and control groups.

Parameters	Intervention group	Control group	Test value	*p*-value
	Mean ± SD/% (n)			
Gender				
Male	62% (8)	57% (8)	0.03^c^	0.8713
Female	38% (5)	43% (6)		
Age (years)	13.23 ± 1.92	14.67 ± 2.08	-1.72^d^	0.0849
IQ	41.92 ± 5.54	40.07 ± 3.32	0.08^d^	0.4519
Place of residence of the pupil’s family				
Village	54% (7)	50% (7)	0.344^c^	0.8528
City	46% (6)	50% (7)		
The pupil’s place of residence during the school year				
Family home	69% (9)	93% (13)	2.40^c^	0.1212
Outside the family home	31% (4)	7% (1)		
Assigned family guardian				
Yes	54% (7)	21% (3)	1.81^c^	0.1789
No	46% (6)	79% (11)		
Significant health and/or family events				
Yes	77% (10)	93% (13)	1.31^b^	0.2531
No	23% (3)	7% (1)		

^a^Pearson’s Chi-square test; ^b^V-square test; ^c^Chi-square test with Yates’ correction; ^d^Mann-Whitney U test.

All research procedures implemented in the project were approved by the appropriate university Bioethics Committee.

### 2.2 Intervention

The original support model for pupils with special educational needs included a program of physical activities based on the Bilateral Integration (BI) School Program, tailored to meet the specific individual needs and capabilities of pupils with moderate intellectual disabilities. The selection of this method as the foundation for the intervention was made with careful consideration. The BI method focuses on promoting correct postural-motor patterns, coordination, alternation, balance, and fine motor skills. Most importantly, it offers a form of physical activity that simultaneously stimulates both movement and cognitive processes. The BI method is grounded in research that demonstrates movement not only fosters physical development but also enhances brain function (27). The BI exercise program aims to develop brain structures responsible for facilitating more efficient and faster information flow between the hemispheres. Previous research suggests that the type of physical activity is crucial in establishing a link between aerobic fitness and the development of executive functions in children with intellectual disabilities. It is thought that, skill-related physical fitness may serve as a stronger predictor of executive functioning (EF) than aerobic fitness. This is based on the hypothesis that learning and development mechanisms, facilitated by skill movements, provide experiences that support cognitive development (24).

The BI program places significant emphasis on the perception, understanding, and using one’s own body. Developing a “body map” — a physical sense of self including awareness of body boundaries, the identification of body parts, and their spatial relationships — is essential for establishing correct posture and enabling alternate use of the body’s left and right sides, as well as upper, lower, front, and back sections in various ways. Accurate data on the body’s current position, its individual elements in relation to each other, and its position within the surrounding space are crucial for proper motor control. The movement sequences employed in the BI method involve specific alternating movements performed in starting positions aligned with the child’s motor development. One system consists of movement sequences of varying lengths (e.g. 24 elements), which the student ultimately performs independently. In teaching movement, polysensory stimulation is used - tactile, visual, and auditory - along with different levels of support provided by the teacher. According to the lesson plans, distractors are minimized at the beginning and cognitive processes are gradually integrated into the reproduction of movement sequences, combining them with the stimulation of the primary sensory channels.

The original modification of the Bilateral Integration School Program is a 26 - week physical activity program in which each student from the intervention group participated in three therapeutic sessions per week. These included two individual sessions (30 minutes each) and one group session (60 minutes) with groups of 6 - 7 pupils). This adaptation was designed to accommodate the special needs and abilities of children and adolescents with moderate intellectual disabilities. The classes followed original lesson plans developed by teachers who were certified Bilateral Integration method therapists. The exercises were aimed at developing and enhancing various skills, including: interhemispheric cooperation; perception and cognitive understanding of one’s own body (“body map”); differentiation of the right and left sides, as well as directions (right/left, up/down, front/back); cooperation within upper/lower, left/right, front/back body dimensions; spatial perception, visual and auditory perception, visual-motor integration and a sense of rhythm overall motor coordination, and bilateral coordination for symmetrical and asymmetrical movements; crossing the body’s midline; posture control, muscle tension distribution, and balance reactions; movement automatization and planning (fine and gross motor skills); fine motor skills such as discriminative sensation and finger separation; sequential and alternating movements, along with improvements in sequential and working memory. A detailed description of the lesson plan creation process, along with specific examples, can be found in the manual published as a product of the grant.

Individual sessions were conducted by trained teachers, each of whom supervised two to three pupils. These sessions provided an opportunity to build a relationship with the child, closely observe their progress, and make necessary adjustments to the. Group sessions, on the other hand, were led by experienced therapists who were also the authors of the lesson plans. These therapists acted as supervisors for the teachers conducting individual sessions. During the group classes, pupils engaged in activities where they observed, compared, and imitated each other. This environment fostered peer motivation, cooperation, mutual acceptance, and the development of self-esteem, self-confidence, and communication skills. This class structure allowed for the continuous oversight of the individual program’s implementation, ensuring adequate support for the teachers, as well as consistent monitoring of each student’s progress throughout the intervention To ensure that all the pupils in the intervention group completed the physical activity program with 100% attendance, make-up sessions were promptly organized for any pupil who missed class due to illness. This approach guaranteed that no student was left behind or missed any critical parts of the program.

### 2.3 Measures

*The Fifth Edition of Stanford–Binet Intelligence Subtests* (SB5) (41). SB5 is a comprehensive, individually administered tests designed for individuals for aged 2 through 85+ (with Polish norms extending to those aged 2-18). It is widely used for both clinical and research purposes to assess general intellectual ability. The test is structured into Verbal and Nonverbal IQ subscales, each comprising five subtests: Fluid Reasoning, Knowledge, Quantitative Reasoning, Visual-Spatial Processing and Working Memory.

These subtests are defined as follows: Fluid Reasoning - the ability to solve verbal and non-verbal tasks using inductive and deductive reasoning; Knowledge – the accumulated general information acquired from various environments, such as home, school, or work; Quantitative Reasoning – the ability to solve numerical problems; Visuospatial Processing – the capacity to recognize patterns, relationships, spatial orientation, and the perception of the whole figures among visual elements; Working Memory – the processes involved in checking, organizing, and transforming information held in short-term memory. Test scores can be converted into Nonverbal IQ, Verbal IQ and Full Scale IQ as well as into ten subtests. All SB5 subtests were administered individually to the pupils by a certified school psychologist in a quiet setting within the school, following the procedural guidelines outlined in the SB5 manual (41). Reliability and validity data for SB5 are available in the interpretive manual (41).

Sociodemographic data were collected from pupils in both the intervention and control groups. This data included variables such as age, sex, place of residence of the pupil’s family, the pupil’s living arrangements during the 2022/23 school year, any significant health and/or family events, and the appointment of a family guardian. Additionally, IQ scores were recorded for all pupils. This information helped ensure the comparability of the two groups and provided context for interpreting the study results.

### 2.4 Data analyses

The analysis of quantitative variables was performed by calculating the mean and standard deviation (SD). Qualitative variables were presented using counts (n) and percentage of each value. The Shapiro-Wilk test was used to assess the normality of data distribution, while Levene’s test was employed to check homogeneity of variance. For variables that did not meet the normality conditions in at least one group, the Mann–Whitney U test was applied to compare the two groups. Pearson’s Chi-square test, Chi-square test with Yates correction, and V-square were used to assess percentage differences between the study groups.

A repeated measures analysis of variance (ANOVA) was conducted to compare the cognitive variables across three measurement points between the two groups. Tukey’s *post-hoc* repeated-measures ANOVA was applied to examine significant group-by-time interactions and to assess changes between the three study terms for the intervention and control groups. Effect sizes (Cohen’s d coefficient) were calculated to describe significant differences in Nonverbal and Verbal SB5 subtest scores, including Nonverbal Visual-Spatial Processing, Working Memory, and Verbal Fluid Reasoning. According to Cohen’s criteria, effect sizes ≥ 0.20 and < 0.50 were classified as small, those ≥ 0.50 and < 0.80 as medium, and those ≥ 0.80 as large (42). All cognitive variables analyses (SB5 subtests scores: Fluid Reasoning, Knowledge, Quantitative Reasoning, Visual-Spatial Processing and Working Memory) were completed using the accumulated raw scores from each subtest, as specified in the test’s procedure manual. The level of statistical significance was set at 0.05 for all statistical analyses. All statistical procedures were performed using the STATISTICA statistical package (TIBCO Software Inc. (2017) Statistica, data analysis software system, version 13. http://statistica.io).

## 3 Results

Thirteen pupils (8 boys, 5 girls) participated in the intervention group, and fourteen pupils (8 boys, 6 girls) participated in the control group. There was no statistically significant difference in gender distribution between the groups (p ≥ 0.05). Both compared groups were also homogeneous in terms of the other sociodemographic variables examined (p ≥ 0.05) (**Table 1**). All participants provided complete data for all test terms (pre-test, post-test, and follow-up testing).

**Table 2** shows the means and standard deviations for all the Nonverbal and Verbal SB 5 subtests (Fluid Reasoning, Knowledge, Quantitative Reasoning, Visual-Spatial Processing and Working Memory) outcome measures for participants in the control and intervention groups at T1, T2, and T3 research dates. The table also presents the results of group-by-time interaction for all cognitive variables.

**Table 2:** Means, standard deviation (SDs), and group-by-time interaction for Verbal and Nonverbal SB 5 subtests.

Outcome measures		Group	Pre-test	Post-test	Follow-up testing	*p*-value repeated measures ANOVA (term x group)
			Mean ± SD			
Nonverbal	Fluid Reasoning	Intervention	9.85 ± 3.69	10.85 ± 4.08	12.54 ± 3.89	0.1114
		Control	10.14 ± 6.31	10.50 ± 5.14	10.00 ± 6.11	
	Knowledge	Intervention	12.54 ± 0.88	12.85 ± 0.99	13.38 ± 0.77	0.8278
		Control	11.50 ± 2.65	11.57 ± 2.10	11.86 ± 2.35	
	Quantitative Reasoning	Intervention	12.15 ± 2.97	13.23 ± 1.09	13.08 ± 1.80	0.5840
		Control	10.43 ± 4.82	10.93 ± 4.05	10.50 ± 5.08	
	Visual-Spatial Processing	Intervention	11.08 ± 2.90	13.54 ± 2.47*	12.38 ± 3.71	0.0303^a^
		Control	10.71 ± 4.79	10.93 ± 5.28	11.07 ± 5.25	
	Working Memory	Intervention	11.62 ± 3.18	14.38 ± 1.94*	14.08 ± 2.06*	0.0231^a^
		Control	11.36 ± 3.99	11.93 ± 4.20	11.43 ± 4.27	
Verbal	Fluid Reasoning	Intervention	8.69 ± 2.43	9.62 ± 2.06	10.46 ± 2.40^#^	0.0164^a^
		Control	6.93 ± 3.83	6.50 ± 4.72	5.43 ± 4.07	
	Knowledge	Intervention	19.54 ± 3.48	22.38 ± 4.37	22.69 ± 4.11	0.1547
		Control	16.21 ± 4.41	18.36 ± 5.37	17.14 ± 5.10	
	Quantitative Reasoning	Intervention	8.31 ± 2.39	9.38 ± 2.60	9.23 ± 2.17	0.2134
		Control	8.57 ± 2.17	8.43 ± 3.74	8.07 ± 3.56	
	Visual-Spatial Processing	Intervention	12.00 ± 2.00	14.38 ± 1.80	13.92 ± 2.69	0.0649
		Control	9.64 ± 4.50	10.21 ± 4.63	10.00 ± 4.88	
	Working Memory	Intervention	10.38 ± 2.84	11.15 ± 2.70	11.62 ± 2.47	0.1074
		Control	8.36 ± 4.09	8.64 ± 4.13	8.57 ± 3.80	

SD, standard deviation; statistical significance p < 0,05; no statistical significance p≥0,05; ^a^significant statistical analysis of variance with repeated measures interaction (groups x time); * statistically significantly different from pre-test; ^#^statistically significantly different from the control group.

### 3.1 Nonverbal measure score

The repeated measures ANOVA revealed a significant group-by-time interaction for the Nonverbal Visual-Spatial Processing score [F(2, 50) = 3.75, p = 0.0303, partial η^2^ = 0.13]. This significant intervention effect was driven by a notable increase in this variable between pre- and post-testing (T1-T2) in the intervention group (p = 0.0018; d = 0,913). No significant changes were observed between the examination dates in the control group.

Similarly, a significant group-by-time interaction was found for the Nonverbal Working Memory score [F(2, 50) = 4.07, p = 0.0231, partial η^2^ = 0.14]. This effect was due to a significant increase in the intervention group between pre- and post-testing (T1-T2; p = 0.0019; d = 1,048) and between post-test and follow-up testing (T1-T3; p = 0.0074; d = 0,918). Again, no significant changes were observed in the control group across the testing periods.

The group-by-time interaction for other Nonverbal variables were not statistically significant: Fluid Reasoning [F(2, 50) = 2.29, p = 0.1114, partial η^2^ = 0.08]; Knowledge [F(2, 50) = 0.19, p = 0.8278, partial η^2^ = 0.01]; Quantitative Reasoning [F(2, 50) = 0.54, p = 0.5840, partial η^2^ = 0.02].

### 3.2 Verbal measure score

The repeated measures ANOVA revealed a significant group-by-time interaction for the Verbal Fluid Reasoning score [F(2, 50) = 4.47, p = 0.0146, partial η^2^ = 0.15]. This significant intervention effect was driven by a significant higher result for this parameter in the intervention group compared to the control group at T3 (p = 0.0073; d = 1,506).

The group-by-time interaction for the remaining verbal variables did not reach statistical significance: Knowledge [F(2, 50) = 1.94, p = 0.1547, partial η^2^ = 0.07]; Quantitative Reasoning [F(2, 50) = 1.59, p = 0.2134, partial η^2^ = 0.06]; Visual-Spatial Processing [F(2, 50) = 2.89, p = 0.0649, partial η^2^ = 0.10]; Working Memory [F(2, 50) = 2.33, p = 0.1074, partial η^2^ = 0.09].

## 4 Discussion

### 4.1 Main findings and study implications

The primary aim of this study was to evaluate the efficacy of an original support model for children and adolescents with special needs, based on the BI method, in enhancing cognitive functioning among pupils with moderate intellectual disabilities. Over a period of 26 weeks, pupils in the intervention group participated in exercise sessions, while those in the control group did not receive any intervention. Cognitive assessments were conducted at three time points: 1) at the beginning of the school year, prior to the intervention; 2) at the end of the school year, following the intervention; and 3) three months post-intervention, after a summer break. It was hypothesized that the intervention would lead to higher cognitive tasks performance in the intervention group compared to the control group.

The results demonstrated improvements in three key areas of cognitive functioning. Statistically significant improvements were observed in the intervention group for nonverbal visual-spatial processing and working memory across subsequent testing points. Additionally, by the third testing term, participants in the intervention group exhibited better outcomes in verbal fluid reasoning compared to those in the control group. In terms of nonverbal visual-spatial processing, participants in the intervention group showed improvement by the second testing term, but there was a slight decline in performance following the summer break. Conversely, the control group’s results remained relatively consistent across all testing terms. Similarly, working memory scores in the intervention group increased significantly after the intervention, and although there was a slight decrease after the holiday break, the scores remained statistically higher than in the first term. No improvement was observed in the control group across any of the testing terms.

The research project was founded on several key assumptions. Firstly, individuals with intellectual disabilities are characterized by a lower general intelligence quotient, however, their internal cognitive profile is varied. For instance, they tend to score lower on verbal subtests compared to non-verbal ones, and exhibit significant difficulties with working memory. Secondly, empirical evidence suggests that children with intellectual disabilities also face movement and motor limitations. They typically demonstrate lower proficiency in fundamental movement skills compared to their typically developing peers. A systematic review highlighted that children and adolescents with intellectual disabilities tend to show deficits or delays in fundamental movement skills, particularly in balance (43).

Thirdly, it has been demonstrated that cognitive functions can generally be improved through education and cognitive training. The effectiveness of such interventions is particularly evident in clinical trials, although sustaining their effects over time can be challenging. The findings of our study align with previous research, such as a meta-analysis on the effectiveness of cognitive training in enhancing working memory among individuals with intellectual disabilities (44). While the original model based on Bilateral Integration primarily involves motor training, it also incorporates cognitive training elements, such as the requirement to remember sequences of movements.

Lastly, research suggests that cognitive functioning can be enhanced through appropriately selected physical activities. Current evidence points to a positive association between physical activity and cognitive functioning. Preliminary studies, similar to ours, have demonstrated that exercise training yields positive effects in both typically developing children (28, 31) and children with intellectual and borderline intellectual disabilities (24). However, given the limited research conducted specifically among individuals with intellectual disabilities, the results of our study should primarily be interpreted in the context of findings from studies on other populations.

It is suggested that participation in regular physical activity enhances memory through changes in the brain, such as increased neurogenesis in hippocampus - an area crucial for learning and memory (40). However, the literature highlights that the specific types and levels of activity that yield the greatest cognitive benefits remain unclear. In our study, we employed exercises that simultaneously required the integration of movement and cognitive skills. The intervention involved tasks targeting both gross motor coordination and fine motor control, aimed at improving balance, postural control, muscle coordination, rhythm, and timing. The exercise program started with a basic motor patterns, progressively increasing in complexity as each student advanced. As students improved, cognitive challenges were incorporated, requiring them to build on previously learned tasks, adjust to new demands, and remember prior instructions. These types of tasks demand significant neurocognitive processing. The improvements observed in this study, attributed to the original modification of the Bilateral Integration School Program, may be due to the layering of cognitive demands onto coordination tasks throughout the intervention.

Working memory capacity is a strong predictor of fluid intelligence (45) and may even represent the most critical factor influencing fluid intelligence (46). Working memory is fundamental to children’s capacity to acquire knowledge and develop new skills. Consequently, many interventions targeting overall cognitive enhancement focus on working memory. Even modest improvements in working memory can have a meaningful impact on children’s academic performance and daily functioning. Interventions like the one analyzed in this study, therefore, offer tangible benefits to the everyday lives of participating children. Visual-spatial processing - which encompasses the ability to recognize patterns, relationships, spatial orientation, and the perception of visual elements as an integrated whole, also holds significant practical and educational implications.

### 4.2 Study strengths and limitations

The present study possesses several notable strengths, including its focus on children with moderate intellectual disability, the inclusion of a control group, the objective measurement of cognitive functions, and the application of an innovative support model based on the Bilateral Integration method tailored for children and adolescents with special needs.

However, the study also has certain limitations. First, the participants selection was based on prior diagnoses of moderate intellectual disability, resulting in variability in the participants’ initial cognitive profile. Future research should aim to recruit a more homogeneous group, although this may prove challenging and result in a smaller sample size. Second, various personal and family-related factors may have influenced participants’ engagement with the intervention and their cognitive test results. While efforts were made to gather information from children, parents, and teachers to identify and exclude participants based on such factors, it is possible that unidentified influences persisted within the final sample, potentially impacting the outcomes. Subsequent studies should prioritize identifying and controlling for potential confounding variables. Finally, although there were no significant differences in age between the groups, the relatively broad age range within the sample (8-18 years), presents a limitation. This issue arose due to the limited number of participating institutions. Future research should encourage collaboration across multiple specialized centers to facilitate the recruitment of larger, more homogeneous study cohorts.

## 5 Conclusion

The present study demonstrated significant cognitive improvements following the intervention, indicating that the proposed model not only enhances physical abilities but also contributes to cognitive development, particularly in nonverbal working memory and visual-spatial processing, among children with moderate intellectual disabilities. Further research is warranted to investigate the underlying mechanisms by which physical activity influences cognitive function in this population. Additionally, future studies should aim to assess the durability of these cognitive benefits and explore effective strategies for sustaining them over the short, medium, and long-term periods.

## Data Availability

The raw data supporting the conclusions of this article will be made available by the authors, without undue reservation.

## References

[1] Maulik PK Mascarenhas MN Mathers CD Dua T Saxena S. Prevalence of intellectual disability: A meta-analysis of population-based studies. Res Dev Disabilities. (2011) 32:419–36. doi: 10.1016/j.ridd.2010.12.018 21236634

[2] King B Toth K Hodapp R Dykens E. Intellectual disability. In: Comprehensive Textbook of Psychiatry, 9th ed. Lippincott Williams & Wilkins, Philadelphia (2009). p. 3444–74.

[3] Hronis A Roberts L Kneebone II. A review of cognitive impairments in children with intellectual disabilities: Implications for cognitive behaviour therapy. Br J Clinic Psychol. (2017) 56(2):189–207. doi: 10.1111/bjc.2017.56.issue-2 28397306

[4] Conners FA Carr MD Willis S. Is the phonological loop responsible for intelligence-related differences in forward digit span? Am J Ment Retard. (1998) 103:1–11. doi: 10.1352/0895-8017(1998)103<0001:ITPLRF>2.0.CO;2 9678225

[5] Danielsson H Henry L Messer D Rönnberg J. Strengths and weaknesses in executive functioning in children with intellectual disability. Res Dev Disabilities. (2012) 33:600–7. doi: 10.1016/j.ridd.2011.11.004 22155533

[6] Memisevic H Sinanovic O. Executive function in children with intellectual disability – the effects of sex, level and aetiology of intellectual disability. J intellect Disabil Res. (2014) 58:830–7. doi: 10.1111/jir.2014.58.issue-9 24206083

[7] Spaniol M Danielsson H. A meta-analysis of the executive function components inhibition, shifting, and attention in intellectual disabilities. J Intellectual Disability Res. (2022) 66:9–31. doi: 10.1111/jir.v66.1-2 34498787

[8] Henry LA MacLean M. Working memory performance in children with and without intellectual disabilities. Am J Ment Retard. (2002) 107:421. doi: 10.1352/0895-8017(2002)107<0421:WMPICW>2.0.CO;2 12323067

[9] Henry L Winfield J. Working memory and educational achievement in children with intellectual disabilities. J Intellectual Disability Res. (2010) 54:354–65. doi: 10.1111/j.1365-2788.2010.01264.x 20236191

[10] Van der Molen MJ Van Luit JEH Jongmans MJ van der Molen MW. Verbal working memory in children with mild intellectual disabilities. J Intellectual Disability Res. (2007) 51:162–9. doi: 10.1111/j.1365-2788.2006.00863.x 17217480

[11] Van Der Molen MJ Van Luit JEH Jongmans MJ van der Molen MW. Memory profiles in children with mild intellectual disabilities: Strengths and weaknesses. Res Dev Disabilities. (2009) 30:1237–47. doi: 10.1016/j.ridd.2009.04.005 19477617

[12] Sajewicz-Radtke U Jurek P Olech M Łada-Maśko AB Jankowska AM Radtke BM. Heterogeneity of cognitive profiles in children and adolescents with mild intellectual disability (MID). Int J Environ Res Public Health. (2022) 19:7230. doi: 10.3390/ijerph19127230 35742482 PMC9223773

[13] Henry LA. The episodic buffer in children with intellectual disabilities: An exploratory study. Res Dev Disabilities. (2010) 31:1609–14. doi: 10.1016/j.ridd.2010.04.025 PMC3025351 20537508

[14] Schuchardt K Maehler C Hasselhorn M. Functional deficits in phonological working memory in children with intellectual disabilities. Res Dev Disabilities. (2011) 32:1934–40. doi: 10.1016/j.ridd.2011.03.022 21570810

[15] Blasi FDD Elia F Buono S Ramakers GJA Nuovo SFD. Relationships between visual-motor and cognitive abilities in intellectual disabilities. Percept Mot Skills. (2007) 104:763–72. doi: 10.2466/pms.104.3.763-772 17688131

[16] Sandfeld Nielsen L Jensen H Skov L. Risk factors of ophthalmic disorders in children with developmental delay. Acta Ophthalmologica. (2008) 86:877–81. doi: 10.1111/j.1755-3768.2007.01158.x 18577186

[17] Maïano C Hue O April J. Fundamental movement skills in children and adolescents with intellectual disabilities: A systematic review. J Appl Res Intellectual Disabilities. (2019) 32:1018–33. doi: 10.1111/jar.12606 31087452

[18] Lahtinen U Rintala P Malin A. Physical performance of individuals with intellectual disability: A 30-year follow-up. Adapted Phys Activity Quarterly. (2007) 24:125–43. doi: 10.1123/apaq.24.2.125 17916913

[19] Maïano C Hue O April J. Effects of motor skill interventions on fundamental movement skills in children and adolescents with intellectual disabilities: a systematic review. J Intellectual Disability Res. (2019) 63:1163–79. doi: 10.1111/jir.12618 31033077

[20] Ma Y Wang L Li M Wang T. Meta-analysis of the effects of exercise programs in improving the balance ability of children with intellectual disabilities. J Intellectual Dev Disability. (2020) 45:144–54. doi: 10.3109/13668250.2019.1632040

[21] Balayi E Sedaghati P Ahmadabadi S. Effects of neuromuscular training on postural control of children with intellectual disability and developmental coordination disorders. BMC Musculoskeletal Disord. (2022) 23:631. doi: 10.1186/s12891-022-05569-2 PMC9250263 35780104

[22] van der Putten AAJ Bossink LWM Frans N Houwen S Vlaskamp C. Motor activation in people with profound intellectual and multiple disabilities in daily practice. J Intellectual Dev Disability. (2017) 42:1–11. doi: 10.3109/13668250.2016.1181259

[23] Campagna J Czyszczon K Little J Selby C Wick L Ferreira D. The physical and psychosocial impact of a school-based running programme for adolescents with disabilities. J Intellectual Disability Res. (2024) 68:181–92. doi: 10.1111/jir.13104 37984471

[24] Hartman E Smith J Houwen S Visscher C. Skill-related physical fitness versus aerobic fitness as a predictor of executive functioning in children with intellectual disabilities or borderline intellectual functioning. Res Dev Disabilities. (2017) 64:1–11. doi: 10.1016/j.ridd.2017.03.001 28288322

[25] Hartman E Houwen S Scherder E Visscher C. On the relationship between motor performance and executive functioning in children with intellectual disabilities. J intellect Disabil Res. (2010) 54:468–77. doi: 10.1111/j.1365-2788.2010.01284.x 20537052

[26] Wuang YP Wang CC Huang MH Su CY. Profiles and cognitive predictors of motor functions among early school-age children with mild intellectual disabilities. J intellect Disabil Res. (2008) 52:1048–60. doi: 10.1111/j.1365-2788.2008.01096.x 18557969

[27] Dalziell A Boyle J Mutrie N. Better movers and thinkers (BMT): an exploratory study of an innovative approach to physical education. Europe’s J Psychol. (2015) 11:722–41. doi: 10.5964/ejop.v11i4.950 PMC4873086 27247688

[28] Dalziell A Booth JN Boyle J Mutrie N. Better Movers and Thinkers: An evaluation of how a novel approach to teaching physical education can impact children’s physical activity, coordination and cognition. Br Educ Res J. (2019) 45:576–91. doi: 10.1002/berj.2019.45.issue-3

[29] Best JR. Effects of physical activity on children’s executive function: Contributions of experimental research on aerobic exercise. Dev Review. (2010) 30:331–51. doi: 10.1016/j.dr.2010.08.001 PMC3147174 21818169

[30] Schmidt M Jäger K Egger F Roebers CM Conzelmann A. Cognitively engaging chronic physical activity, but not aerobic exercise, affects executive functions in primary school children: A group-randomized controlled trial. J Sport Exerc Psychol. (2015) 37:575–91. doi: 10.1123/jsep.2015-0069 26866766

[31] Dalziell A Boyle J Mutrie N. Better movers and thinkers (BMT): A quasi-experimental study into the impact of physical education on children’s cognition – A study protocol. Prev Med Rep. (2015) 2:935–40. doi: 10.1016/j.pmedr.2015.10.004 PMC4721461 26844172

[32] Sibley B Etnier J. The relationship between physical activity and cognition in children: A meta-analysis. Pediatr Exercise Science. (2003) 15:243–56. doi: 10.1123/pes.15.3.243

[33] Corbin C Pangrazi R Franks B. Definitions: health, fitness, and physical activity. President’s Council Phys Fitness Sports Res Digest. (2000) 3.

[34] Diamond A. Close interrelation of motor development and cognitive development and of the cerebellum and prefrontal cortex. Child Dev. (2000) 71:44–56. doi: 10.1111/cdev.2000.71.issue-1 10836557

[35] Koziol LF Budding D Andreasen N D’Arrigo S Bulgheroni S Imamizu H. Consensus paper: The cerebellum’s role in movement and cognition. Cerebellum (London England). (2014) 13:151–77. doi: 10.1007/s12311-013-0511-x PMC4089997 23996631

[36] van der Fels IMJ Te Wierike SCM Hartman E Elferink-Gemser MT Smith J Visscher C. The relationship between motor skills and cognitive skills in 4-16 year old typically developing children: A systematic review. J Sci Med Sport. (2015) 18:697–703. doi: 10.1016/j.jsams.2014.09.007 25311901

[37] Holzapfel SD Ringenbach SDR Mulvey GM Sandoval-Menendez AM Cook MR Ganger RO. Improvements in manual dexterity relate to improvements in cognitive planning after assisted cycling therapy (ACT) in adolescents with down syndrome. Res Dev Disabil. (2015) 45–46:261–70. doi: 10.1016/j.ridd.2015.08.003 26280691

[38] Singh AS Saliasi E van den Berg V Uijtdewilligen L de Groot RHM Jolles J. Effects of physical activity interventions on cognitive and academic performance in children and adolescents: a novel combination of a systematic review and recommendations from an expert panel. Br J Sports Med. (2019) 53:640–7. doi: 10.1136/bjsports-2017-098136 30061304

[39] Cotman CW Berchtold NC Christie LA. Exercise builds brain health: key roles of growth factor cascades and inflammation. Trends Neurosciences. (2007) 30:464–72. doi: 10.1016/j.tins.2007.06.011 17765329

[40] van Praag H. Neurogenesis and exercise: past and future directions. Neuromol Med. (2008) 10:128–40. doi: 10.1007/s12017-008-8028-z 18286389

[41] Roid G Jurek P Olech M Sajewicz-Radtke U Radtke B. Skale Inteligencji Stanford-Binet, Edycja Piąta. (2017), Podręcznik techniczny.

[42] Cohen J. Statistical Power Analysis for the Behavioral Sciences. 2nd ed. Hillsdale, NJ: Lawrence Erlbaum Associates (1988).

[43] Maïano C Hue O Morin AJS Lepage G Tracey D Moullec G. Exercise interventions to improve balance for young people with intellectual disabilities: a systematic review and meta-analysis. Dev Med Child Neurology. (2019) 61:406–18. doi: 10.1111/dmcn.14023 30230530

[44] Danielsson H Zottarel V Palmqvist L Lanfranchi S. The effectiveness of working memory training with individuals with intellectual disabilities – a meta-analytic review. Front Psychol. (2015) 6:1230. doi: 10.3389/fpsyg.2015.01230/abstract PMC4538918 26347692

[45] Kane MJ Hambrick DZ Conway ARA. Working memory capacity and fluid intelligence are strongly related constructs: comment on ackerman, beier, and boyle (2005). psychol Bulletin. (2005) 131(1):66–71. doi: 10.1037/0033-2909.131.1.66 15631552

[46] Conway ARA Cowan N Bunting MF Therriault DJ Minkoff SRB. A latent variable analysis of working memory capacity, short-term memory capacity, processing speed, and general fluid intelligence. Intelligence. (2002) 30:163–83. doi: 10.1016/S0160-2896(01)00096-4

